# Design and implementation of student management system of integrated programmable device programming system

**DOI:** 10.1038/s41598-024-62844-z

**Published:** 2024-05-24

**Authors:** Huijin Huang, Benyuan Li

**Affiliations:** Guangdong Polytechnic of Science and Technology, Zhuhai, 519090 China

**Keywords:** Student management system, Neural networks, Programming system, System test, Computational science, Computer science

## Abstract

With the continuous growth and development of society, the reform of higher education is gradually taking shape, and colleges and universities are taking more and more responsibilities in promoting education. The main element of university management is the student management system, which is very important to the development of the university. Under the objective environment of colleges and universities seeking to expand the scale of running schools and rapid economic and social development, the current student management system has been unable to meet the various needs of contemporary students. The integration of programmable device programming systems offers a student management system distinct advantages in terms of reliability, flexibility, and user-friendly operation. This study focuses on developing an effective and affordable student management system by incorporating a programmable device programming system. To evaluate the system's performance, this paper suggests the utilization of a BP neural network, renowned for its high nonlinear approximation capabilities and effectiveness in handling complex nonlinear functional relationships. The experimental findings highlight a significant contribution, demonstrating that the system achieved a throughput of 180 times per second, with a maximum CPU utilization of 99%. Notably, the system's stability exceeded 95%, contrasting sharply with the traditional student management system's stability at a mere 65%. These results underscore the substantial contribution of the proposed system, showcasing its enhanced stability compared to conventional student management systems.

## Introduction

As school institutions continue to expand, managing students becomes more challenging. Based on the guiding principles of student management and using advanced knowledge and concepts from other countries, these concepts are naturally integrated into the operational characteristics of Chinese universities to build a complete student management platform and a set of core student process management^1^. Managers can achieve the unified, effective, interconnected, and information-sharing whole process management of college students through the student whole process management platform.

With the basic construction of college campus network, many informatization and digital teaching resources are continuously developed. The management informatization process of school administrative office is also accelerating, and managing the daily study and life of school teachers and students is very important to the digital construction of campus^2^. Through the use of informatization and digital tools, the efficiency of school management and teaching has been improved, and colleges and universities have recently expanded their capital expenditures for campus digitalization^3^. The development of various educational and teaching information management systems, as well as the installation of campus information and communication hardware and networks, cost a lot of money, especially in terms of management.

After the official operation of the student management system, it should be able to reduce the workload of relevant managers and greatly improve the efficiency of work. The characteristics of student management are embodied in the following three aspects:

1. The application of database means in the system makes the data information more complete, reduces the occurrence of duplicate data, and effectively realizes the data sharing among multiple departments and users.
2. The use of student management system makes the management of the school more standardized, while human factors are greatly reduced. It affects the overall management of the school to be more fair and transparent.
3. Due to the need of the country to develop skilled talents and the policy orientation of the school, the number of students in the school has been increasing, and the original resources of the school have become tense. The use of the student management system can make the existing resources of the school reasonable use.

This study makes a notable contribution in the realm of student management systems by proposing the development of an effective and budget-friendly solution through the incorporation of a programmable device programming system. The evaluation of the system's performance employs a BP neural network, recognized for its exceptional nonlinear approximation capabilities and efficacy in handling intricate nonlinear functional relationships. The experimental outcomes reveal a substantial advancement, as the system achieves a remarkable throughput of 180 times per second, coupled with a peak CPU utilization of 99%. Significantly, the system's stability surpasses 95%, in stark contrast to the traditional student management system's modest stability of 65%. These findings emphasize the meaningful impact of the proposed system, highlighting its superior stability when compared to conventional student management systems.

## Related work

The management of students must meet higher standards as the nation and society place increasing emphasis on the caliber of education. Bizarria et al.^4^ examined the crucial factors for the efficient growth of colleges and universities. The academic preparation of students and the preparation of teachers are all included in student management work, and it is carried out in the configuration of knowledge and experience. Santana et al.^5^ presented management methodology as an alternative to teaching practice and software project management. He surveyed students at a university. He attempted to analyze students' achievements in management practice and their perceptions of learning methods, showing both positive and negative aspects through this experience. In order to obtain the movement track information of college students, Ding^6^ uses Smoiler to collect the original location information of students on the student mobile phone client, uses Spark to extract the offline original data of students and store it in Hive, and then filters, analyzes and calculates the data stored in Hive and stores it in the relational database. Finally, it visualizes the data to provide decision support for college administrators. He^7^ designed a web-based employment information management system for vocational college students. The system hardware is designed with STM32F10X embedded processor. The system web architecture is divided into three layers: the presentation layer, the control layer and the data access layer. It provides convenience for information display and employment guidance, completes, receives and processes tasks from user requests, completes the database design, and reasonably maintains data resources. Through the test and analysis of the student employment information management system, this research has verified that the system is fast and accurate, and can reasonably manage student employment information. From the standpoint of the public perception of students and educational institutions, Jogunola and Varis^8^ discussed the quality of higher education and the management practices that gauge it. The study's findings would aid colleges in managing and growing more effectively in the future. According to academics, student management systems are becoming more prevalent in colleges and universities due to the advancement of information technology. The original system has low work efficiency and is not very flexible. The non-linear nature of artificial neural networks allows for their versatile application in diverse domains. Li^9^ exemplified this adaptability by employing neural network theory in the evaluation of electronic library quality. He devised a library quality assessment model based on the neural BP network, effectively addressing subjectivity and ambiguity inherent in traditional assessment methods. This facilitated a scientifically rigorous evaluation of electronic resources. Turning to the domain of regional sustainable development, Wu et al.^10^ emphasized the pivotal role of land ecological security assessment. Their work involved enhancing the BP neural network through the development of an assessment index method, incorporating Genetic Algorithm (GA). This integration proved effective in overcoming challenges such as slow convergence speed and susceptibility to local minima when applying the BP neural network to land ecological security evaluation. In the realm of uncertain delay neural networks, He et al.^11^ contributed by presenting conditions ensuring the presence of a required state estimator. The study demonstrated the ability to develop a robust state estimator for such neural networks by resolving linear matrix inequalities. Jiang and Wang^12^ proposed an approach utilizing the Analytic Hierarchy Process (AHP) and particle swarm optimization in conjunction with BP neural networks. Through continuous adjustment of model parameters using the BP-based neural network assessment model, they achieved scientifically and administratively sound evaluation results across various industries. While scholars have successfully applied neural networks for evaluations in diverse sectors, there is a notable gap in explaining the application of neural networks to the evaluation of student management systems. This underscores the need for further exploration and innovation in leveraging neural networks for the improvement of student management system assessments.

## Student management system based on artificial neural network

### Basic structure of programmable controller

**Figure 1:**
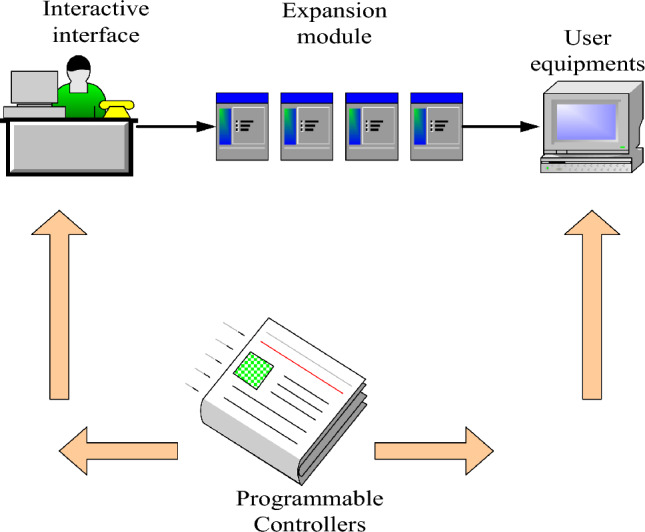
Schematic diagram of programmable controller.

As the central processing unit of equipment, programmable logic controller (PLC) has been widely used in the automatic management of many manufacturing processes and equipment. It has the characteristics of simple operation, simple understanding and high reliability^13^. It has developed into the most well-known, important and commonly used industrial control equipment, and it is also one of the key pillars in the field of contemporary industrial automation control systems^14^. The schematic diagram of the programmable controller is shown in Fig. 1:

As depicted in Fig. 1, the integration of the programmable device programming system into the student management system facilitates the implementation of intelligent reasoning and decision-making capabilities in relay protection. This integration showcases the reliability and efficiency of control technology, establishing a crucial foundation for the practical implementation of effective management systems. Operating within a safe range, this system allows for the monitoring and control of power system equipment and lines. It enables the timely detection and handling of abnormal conditions, such as faults, thereby preventing unnecessary losses. This approach serves as a decision-making basis, contributing to the safe, reliable, and stable operation of the management system^15,16^.

Most of the original management systems are assessment-related application systems. The specific work done by various colleges and universities, such as enrollment work, entry work, management of students during school, and application of information technology have not improved the daily management of students and other key tasks. These systems can increase teacher productivity and improve school conditions. This approach can significantly increase productivity in every department. It enables university administrators to gain a faster, more comprehensive, and more appropriate view of student status and the progress of various programs.

This paper uses JavaEE technology. It uses MyEclipse8 as the development environment, and uses the sqlserver2008 database design to develop a complete process management system for college students^17^. This paper focused on the deployment strategy and design scheme of student whole-process management.

### System requirements

The non-functional requirements of the entire college student process management system constructed in this paper mainly focus on determining whether the system can meet the user's access requirements during operation^18^. It mainly includes the amount of user access data received in real time when the system is running, the response time and response time of the system, the ability to handle emergency situations, and other system performance criteria. The following is a description of the non-functional requirements of the system.

1. Performance requirements. The data information of the entire higher education management system is transmitted through the facilities and lines of the school network. Therefore, if the network is normal and the line is disconnected, the response speed of the system to customer requirements should not exceed 3 s. When a network failure occurs, the system should provide the customer with appropriate information about the alarm, help the customer to find the problem in time and locate the fault site quickly, so as to complete the operation required by the customer.
2. Reliability specification. The operation of the whole process management system for college students needs to input relevant student data, and then send the data to the background database of the system for data processing and analysis. Therefore, when the system is in the input stage, it is necessary to identify and evaluate the security and reliability of the student data information input by the user, and filter out any student data information that does not meet the security standards.
3. Ease of use is required. When developing and designing the whole process management system for college students, the user-friendliness of human–computer interaction should be fully considered, because the computer proficiency of system users (operators) varies. The entire process management system created and designed for college students must ensure that the operation process of each function is simple and clear, and improve the usability of human–computer interaction. Furthermore, in order to limit operator errors, specific instructions for the operational phases need to be provided to prevent system users from erroneously operating the system.

**Figure 2:**
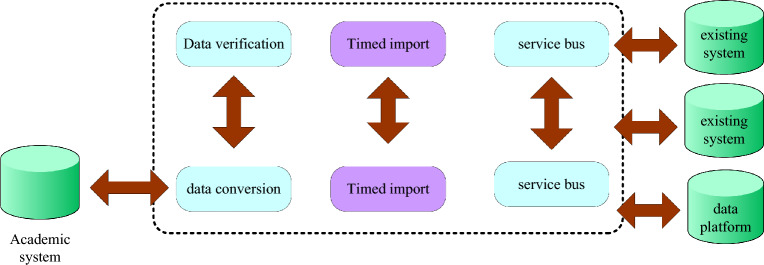
Schematic diagram of integration between systems.

Currently, all colleges and universities have successfully concluded a phase of informatization construction, encompassing not only the enhancement of network and host infrastructure but also the development of several high-quality application systems. To seamlessly access the comprehensive data repositories established across various universities, these application systems must integrate with the students' end-to-end process management and analysis system. Illustrated in Fig. 2^19^, this system is intricately linked with existing systems at three levels: interface, function, and data integration. Notably, this article adopts the industry-standard service-oriented architecture, commonly referred to as SOA architecture, to ensure a cohesive and efficient integration of these diverse components.

Figure 2 illustrates how to implement the user rights management mechanism for the entire student flow and analysis management system and how to successfully support the implementation of a particular application function. It is necessary to develop user functions and data rights management authorization mechanisms. When using the student management system, there are very strict instructions to manage the user rights of the system, the system usually authenticates, authorizes and manages the authorization of system users^20^.

**Figure 3:**
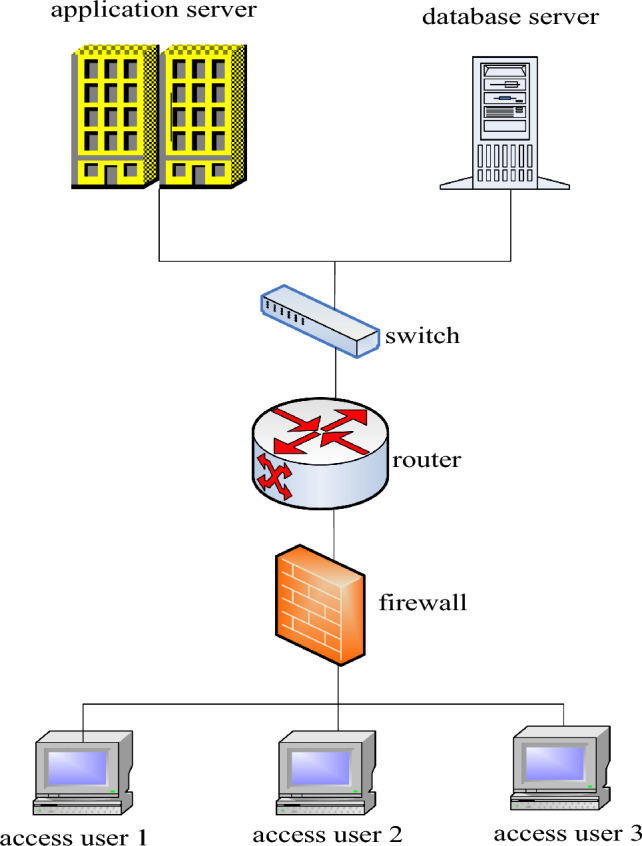
System network topology diagram.

For example, the advanced file management system service can successfully realize flexible file classification during system operation, and provide corresponding file management services for the basic trading platform of the application system. It improves the efficiency of system protocol b access management to a certain extent^21^, as shown in Fig. 3:

As shown in Fig. 3: The main responsibility of the data resource layer is to store persistent application system data and information resources. In the storage process, the data source of the system is permanently stored in the form of database, address server and file system. Labor procedures are required for every activity in a college student management system, such as reviewing and approving student misconduct. Students manage and analyze systems throughout the process to support business processes using a workflow engine. By manually dragging and dropping objects, users can simultaneously re-customize and sequence business processes^22^.

### System function realization

1. Function realization of student information management module

**Figure 4:**
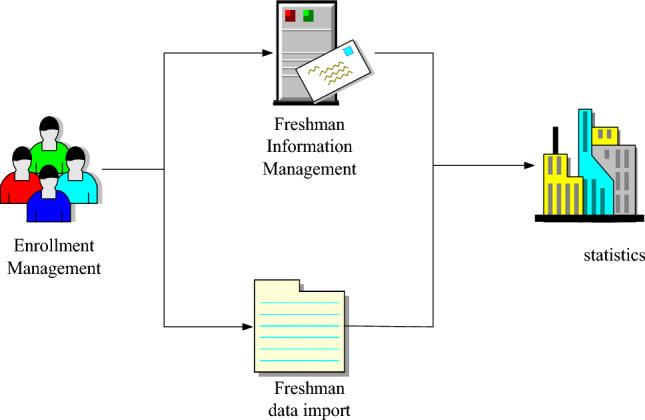
Student basic information management sub-module.

The Student Information Report module subscription feature allows users to view existing system data. It supports fuzzy data subsidies and can enforce conditional subsidies based on the data range of interest to the system users. New functions include adding new parts of data to the database, and modifications to existing data records are called modify functions. Existing system data records can be deleted using the delete function. In addition to the system pages, specific functions would be discussed in detail. An example of the student basic information management sub-module is shown in Fig. 4:

As shown in Fig. 4: After filling in the new page and modifying the page, the person clicks "Save" to save the data, and closes the page after saving. In addition to the questioning and storage functions of students' basic information, the student's basic information report module also provides statistical analysis functions. A simple statistical analysis of students' basic information can be performed in the Statistics sub-module.

(2) Realization of counselor management function

Throughout the academic journey, the office's counselors and students engage in a collaborative process of reviewing job applications submitted by students. The initial phase involves instructor reviews, where instructors input assessment results, comments, and other pertinent data for each position applied to by the student. Following the entry of review information, all authorization result records are then submitted to the Student Office for approval.

Within the management module, three primary sub-modules contribute to efficient functioning. These include teacher information maintenance, teacher class management, and teacher statistical analysis. The counseling information maintenance sub-module takes on the responsibility of overseeing and preserving various counselor data. This encompasses essential information such as basic counselor details, local contacts, political affiliation data, family communication data, family member data, accommodation data, and other relevant information. This comprehensive approach ensures the effective management of counselor-related data for streamlined student support and guidance.

### Algorithm based on BP neural network

Artificial neural network is a nonlinear system with high learning ability, good adaptability and self-organization ability^23^. For complex unknown coefficients with different sources, a highly nonlinear mapping relationship can be achieved without creating mathematical models for artificial neural networks^24^. Each neuron stores different weights and closing value groupings that make up the network information. It refers to knowledge extracted from data by network training and used to evaluate or predict outcomes for similar factors. Thus models for quantitative and qualitative evaluation can be developed, and artificial neural networks can be used to evaluate systems.

**Figure 5:**
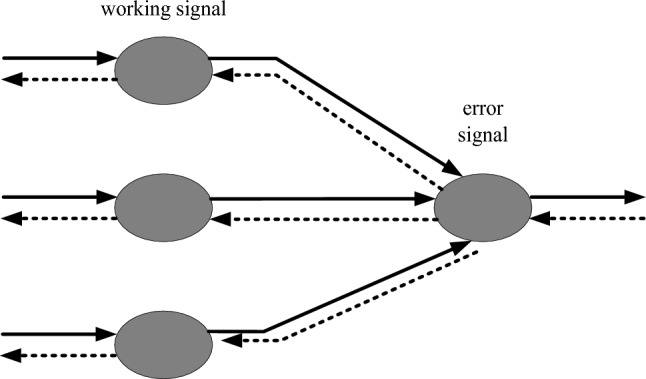
BP network learning process.

The two mechanisms that form the learning process of the BP network are the first dissemination of information and the second dissemination, as shown in Fig. 5:

As shown in Fig. 5: If the output layer cannot produce the desired output, the error signal should be transmitted to the backward distribution. The weights of each layer for signal forward propagation and error back propagation are repeatedly changed in this way. The network is trained through a continuous weight adjustment process. The process should continue until the network output error is acceptable or a predetermined number of times are obtained.

The output error E occurs when the network output is not equal to the expected output, which is defined as:

1 $$ E = \frac{1}{2}\left( {d - o} \right)^{2} = \frac{1}{2}\sum\limits_{k = 1}^{l} {\left( {d_{k} - o_{k} } \right)}^{2} $$

Extending the above error definition to the hidden layer, there is Formula 2:

2 $$ E = \frac{1}{2}\sum\limits_{k = 1}^{l} {\left( {d_{k} - f\left( {net_{k} } \right)} \right)}^{2} $$

The following calculation makes it evident that the network input error depends on the $d_{k}$ of each layer, so changing the weight would affect the error E. The idea of weight adjustments is, of course, to continually reduce error, therefore the adjustment weight should be inversely proportional to the negative error gradient, or formula 3:

3 $$ \Delta w_{jk} = - \eta \frac{\partial E}{{\partial w_{jk} }},j = 0,1,2,...,m;k = 1,2,...,l $$

The constant $\eta$ is a scaling factor that reflects the learning rate during training, while the negative sign in the formula denotes gradient descent. It is envisaged that artificial neurons would have a more general transfer function that would be used to change the network input received by the neuron to expand the applicability of the system. The excitation function is denoted by the symbol F:

4 $$ f\left( a \right) = \left\{ {\begin{array}{*{20}c} {1,a \ge 0} \\ {0,a \prec 0} \\ \end{array} } \right. $$

A vector of samples must be entered continuously, and the training weights must be adjusted based on the difference between the actual and expected outputs. Finally, the performance indicators must be adjusted to meet the required standards in order to make the network meet the specific application requirements. This process needs to be repeated.

### System evaluation model based on BP neural network

Before making comparisons, the estimated values of each indicator have different measurement techniques and need to be standardized. It is necessary to use training and testing samples to standardize the evaluation values of the metrics. The choice of normalization function is critical when performing data normalization. The ranges must be distinct and the normalization function must be strictly monotonic. In this paper, the system evaluation index data is standardized, and the input data range is [0,1], which is Formula 5:

5 $$ b_{ij} = \frac{{a_{ij} - \min a_{ij} }}{{\max a_{ij} - \min a_{ij} }},i = 1,2,...,n,j = 1,2,...,p $$

Among them, $a_{ij}$ represents the data value of the jth attribute of the ith evaluation object, and the maximum and minimum values of all data in the attribute are represented by $\max a_{ij}$ and $\min a_{ij}$.

After normalizing the evaluation index values, the input value and output value range from 0 to 1. Therefore, the transfer function and the logsig function of the hidden layer and the Tansig output layer are the same, which is Formula 6:

6 $$ \log sig\left( a \right) = \frac{1}{{1 + e^{ - a} }} $$

The mathematical relationship between the input layer and the hidden layer:

7 $$ b_{j} = \tan sig\left( {net_{j} } \right) $$

$net_{j}$ indicates the input of the jth neuron in the hidden layer, and $b_{j}$ indicates the output of the jth neuron in the hidden layer.

The BP neural network model's structure is mostly dependent on the number of hidden layers. The hidden layer, output layer, and input layer neurons are all counted in the second stage. The single hidden layer BP neural network has a very high nonlinear mapping capability. The general formula for figuring out the hidden layer is Formula 8:

8 $$ p = \sqrt {n + m} + a $$

The mathematical relationship between the hidden layer and the output layer is equal to Formula 9:

9 $$ O_{k} = \log sig\left( {net_{k} } \right) $$

Input $O_{k}$ is the input of the kth neuron in the output layer, and output value $net_{k}$ is the output value of the kth neuron.

The sigmoid function is a crucial excitation function that is frequently employed as the excitation function, regardless of whether a neural network has been used for classification, such as Formula 10:

10 $$ b = f\left( u \right) = \frac{1}{{1 + E^{ - \lambda u} }} $$

^-λu^ represents the parameters of the Gaussian function, and the Gaussian function is also an important one of the excitation functions. The expression for the Gaussian function is Formula 11:

11 $$ b = f\left( u \right) = e^{{ - \frac{{u^{2} }}{{\sigma^{2} }}}} $$

The parameter $\sigma$ is called the width or expansion coefficient of the Gaussian function. The larger the value of $\sigma$, the flatter the function curve; the smaller the value, the narrower the function curve.

If the purpose of correcting the neuron weight is to minimize the scalar function, and the actual weight of the neuron is $W\left( k \right)$, the formula for correcting the weight at the next moment should be Formula 12:

12 $$ W\left( {k + 1} \right) = W\left( k \right) + \Delta W\left( k \right) $$

Among them, $\Delta W\left( k \right)$ includes the current correction amount. Formula 13 is clearly expected for each correction:

13 $$ J\left[ {W\left( {k + 1} \right)} \right] \prec J\left[ {W\left( k \right)} \right] $$

If the function of a neuron is linear and its excitation function is a fixed limit function, the neuron becomes a single neuron perceptron. Discrete perceptual learning rules are also supervised learning algorithms, which is the name of a perceptual learning rule for neurons. The neuron excitation function is a symbolic function, the perceptron discrete learning rule is applied, and the weight is adjusted as Formula 14:

14 $$ \Delta W\left( k \right) = e\left( k \right)A $$

The Hebb learning rule is an uncontrolled learning algorithm that is usually used in self-organizing networks or networks that extract functions. According to Hebb's rule, the current entry of the neuron is equal to Formula 15:

15 $$ A = \left[ {a_{1} ,a_{2} ,...,a_{n} } \right]^{T} $$

The weight correction formula of neurons is:

16 $$ W\left( {k + 1} \right) = W\left( k \right) + \eta bA $$

Neuron weights usually acquire random values close to zero, and the firing function can take any form.

## Experiment of student management system integrating programming system

### Evaluation experiment of student management system based on neural network

During the initial phases of model application, the BP neural network is modeled and implemented using Matlab R2015b. The modeling process leverages the functions and simulation tools provided by Matlab. Subsequent to the completion of BP neural network training, a network test is conducted using 50 data points from the test sample set. The Table 1 displays the evaluation results of a system across different data points, spanning from 10 to 50. The "Data" column enumerates the specific input values used for testing, while the "Actual Output" column reveals the system's generated outputs during testing. In parallel, the "Expected Output" column outlines the anticipated or desired output corresponding to each data point. The "Error" column quantifies the disparity between the actual and expected outputs, calculated as the absolute difference. For example, when the data is 10, the error is computed as 0.02, reflecting the deviation between the actual output (0.87) and the expected output (0.85). This detailed breakdown offers insights into the system's performance, aiding in the assessment of accuracy and identification of potential areas for refinement within the model. The comprehensive evaluation findings of the system are then obtained and documented.

**Table 1:** Comprehensive evaluation results of the system.

Data	Actual output	Expected output	Error
10	0.87	0.85	0.02
20	0.88	0.90	0.01
30	0.90	0.89	0.01
40	0.93	0.92	0.01
50	0.96	0.93	0.03

**Figure 6:**
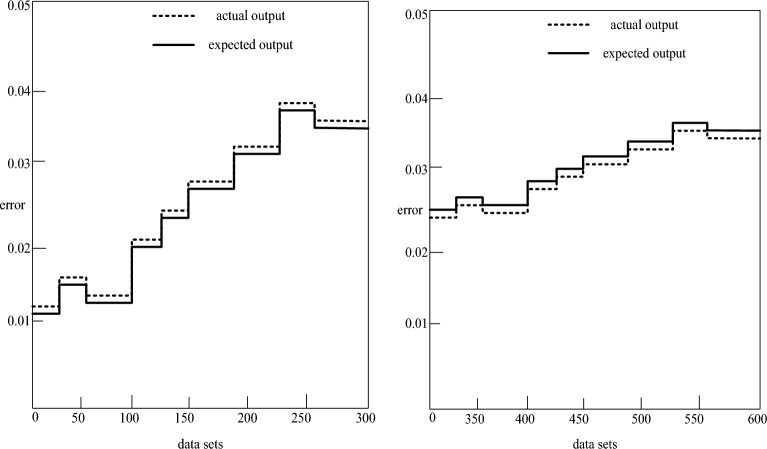
Comparison of expected and actual results.

The experimental findings, as depicted in Table 1, underscore the remarkable evaluation outcomes achievable through the utilization of the BP neural network to assess the system. This approach introduces a novel perspective for system review, enhancing the speed and effectiveness of the evaluation process. The results obtained from the evaluation network model carry significant guiding implications for the assessment of the student management system. To establish the scientific validity of these conclusions, experiments were meticulously conducted on 600 groups of student management data. The comparison of expected results with actual results, as illustrated in Fig. 6, provides a visual representation of the experimental validation process, reinforcing the robustness and reliability of the findings.

Figure 6 illustrates how the training and prediction accuracy of the student management system assessment model based on BP neural network are both fully within the allowed range, making it a workable prediction model. The established neural network has an excellent evaluation effect and may be used for the following evaluation of the student management system since the difference between the network's actual output and the expected output is extremely minimal. It demonstrates that the developed BP neural network is capable of fully assimilating professional judgment and actual student management, enabling it to carry out a more scientific examination of test results. Additionally, it encourages the usage of BP neural network to assess the viability and efficacy of the student management system.

### Student management system performance test

After all software is created, it should be tested to identify any systemic issues. Software testing should check for compatibility and lack of functionality of the software and its usefulness, it must be tested under various browser settings. Since the management system established in this paper is based on an operational web application platform, the requirements for the system testing environment are relatively low, requiring personal computers, network resources and ready-made test cases. The software and hardware environment of the test system are shown in Table 2:

**Table 2:** Software and hardware environment for system testing.

Hardware resources	Name/type	Remark
Web server	CPU	Intel Xeon E5-2683 v3
	Memory	6 GB
	Hard disk	512 GB
	Operating system	Windows 7
Database server	Memory	6 GB
	Hard disk	512 GB

As shown in Table 2: The system database may run under abnormal conditions, such as when system users access the system too frequently, which would allow the system server to accept a large number of current user access requirements. The intermediate application service layer continuously tries to restore the connection to the background database to avoid the above situation and respond to the client's access demand instructions.

The performance test of the entire higher education management system can verify that the system has good stability and robustness during operation. The tests are shown in Table 3:

**Table 3:** Summary of test situations.

Serial number	1	2	3
Number of users (person)	60	70	80
Execution time/ms	15.67	16.82	20.55
Server average available memory (M)	3655	3600	3580
Server memory utilization	More stable	More stable	More stable
Whether there is an error or abnormality in the system	None	None	None

As shown in Table 3: The number of users in the system experiments outlined in this paper are 60, 70, and 80, respectively. As the number of user’s increases, the memory also drops, but the drop is not significant, from 3655 M in the first test to 3580 M in the third test. Furthermore, the system did not report any errors or anomalies in the three tests.

**Figure 7:**
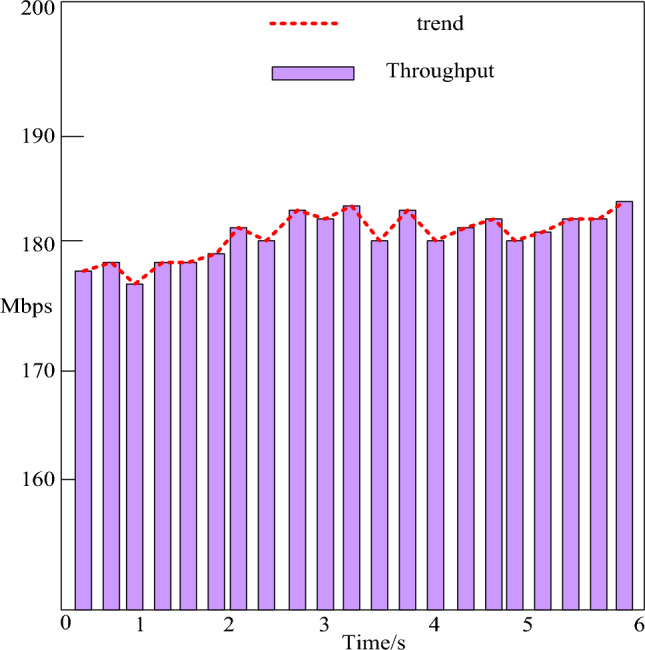
Hits per second-throughput analysis.

This paper analyzes the click rate-throughput of the system, as shown in Fig. 7:

As seen in Fig. 7, the chosen users log in to the college student full process management system. It is lined with the specific scene setting criteria to test the system's functionality and carry out relevant tasks. The analysis shows that the system runs smoothly, the performance is basically stable, and no errors are found in the whole performance testing process. According to the entire testing process, as the number of hits per second increases, the throughput increases at an average rate of 180 hits per second. If the hit rate per second drops, the associated throughput would drop. Interventions per second and acceptability tend to be about the same, which is a good sign that the server is responding to client requests while the system is running.

**Figure 8:**
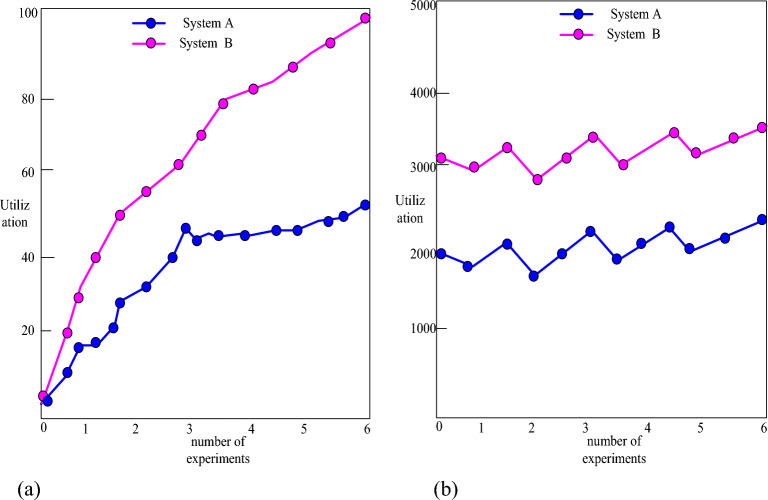
Windows resource usage.

In order to prove that the system proposed in this paper has more advantages, this paper compares the traditional student management system (system A) and the system designed in this paper (system B), as shown in Fig. 8:

As shown in Fig. 8: From Figure (a), it can be seen that both the physical memory and the system's CPU can be used in combination with a maximum utilization rate of 99%. This test shows that the server's memory is larger than the average physical memory, within the typical range. It can be seen from Figure (b) that System B can run stably under the condition of simultaneous access by multiple users.

On the basis of analyzing the current test data and test effect, this paper proposes a whole process management system for college students, and tests it from different angles. In terms of system data security, system function module requirements, and system operation performance, it basically meets the requirements of the initial stage of system development. In actual use, the system works well, and all of its features can accommodate college students' needs throughout the management process.

Table 4 displays the satisfaction scores of the seven teachers who used both the traditional student management method and the approach proposed in this paper.

**Table 4:** System satisfaction.

Teachers	System A (%)	System B
1	65	95
2	63	97
3	61	96
4	59	98
5	62	99
6	65	96
7	58	95

As shown in Table 4: System B has 99% stability at its highest point and 95% stability at its lowest point. While System A has 65% stability at its highest value and 58% stability at its lowest value. Although the development cycle of college student whole management system is short, the actual operation effect of the system can adapt to the daily management requirements of student whole management. This method can make it easier for administrators of the whole process management system of college students to manage the whole process of online students, which greatly improves the scientific management level of the whole process of college students. The comparative performance of two systems, System A and System B, across seven distinct categories of teachers. In System A, the percentage scores achieved by teachers range from 58 to 65%, with the lowest score recorded in category seven and the highest in categories one and six. Conversely, in System B, the percentage scores exhibit a wider range, from 95 to 99%, showcasing a generally higher performance level compared to System A. Category five stands out as the highest-scoring group in System B, achieving 99%. Notably, System B consistently maintains higher scores across all categories when juxtaposed with System A, reflecting its superior performance in this dataset. This table serves as a succinct visual representation, allowing for a quick comparison of the performance metrics between the two systems across various teacher categories.

## Conclusion

The pivotal phase in educational informatization is campus informatization, where the integration of information architecture with education and information technology yields substantial benefits. This integration extends the temporal and spatial dimensions of educational institutions, enhancing the efficiency of teaching and elevating the standards of decision-making, management, and student training. The rapid growth of colleges and universities in China, coupled with governmental efforts to advance higher education, has underscored the increasing importance of effective management within the Chinese education system. In the current competitive economic landscape, the imperative to enhance management quality at educational institutions becomes crucial for their sustained success. Addressing challenges such as scale, faculty shortages, and other resource limitations is essential for maintaining and elevating the quality of teaching. Various results are obtained for evaluation as reported in Table 1, the error between actual and expected outcomes is about 0.01 to 0.03. For various datasets. The stability is checked in term of time consumption. Three datasets are discussed with time per minute is 15.67, 16.82 and 20.55 with a server average available memory 3655, 3600 and 3580, respectively. Every teacher is evaluated in two different systems. In system A the satisfaction level is form 585 to 65%, while in system B the satisfaction level is 955 to 99%. The creation of a student management system, coupled with the integration of a programmable device programming system, represents a contemporary approach to university management. Experimental findings suggest that this integrated system not only exhibits superior throughput but also demonstrates heightened stability. However, it is acknowledged that the experiment's limitations, such as its simplicity and the absence of a college or university setting, impact the precision of the conclusions. Future studies are thus encouraged to enhance the experiment's robustness by increasing the sample size and extending the scope to college and university environments.

## Data Availability

The experimental data used to support the findings of this study are available from the corresponding author upon request.
